# Machine Perfusion in an Ageing Population - Results From a German, National Survey Among Transplant Centers

**DOI:** 10.3389/ti.2025.15681

**Published:** 2025-12-19

**Authors:** Mohammed Ibrahim, Judith Dechantsreiter, Philip C. Müller, Michele Finotti, Mikolaj R. Kowal, Jonel Ngjelina, Silvio Nadalin, André L. Mihaljevic, Philipp Kron

**Affiliations:** 1 Department of General, Visceral and Transplant Surgery, University Hospital Tübingen, Tübingen, Germany; 2 Department of Surgery, Clarunis University Digestive Health Care Center, University Hospital Basel, Basel, Switzerland; 3 Leeds Institute of Medical Research, Faculty of Medicine and Health, University of Leeds, Leeds, United Kingdom

**Keywords:** ECD grafts, kidney, liver, organ utilization, transplantation

## Abstract

Machine perfusion (MP) is rapidly emerging as an alternative to static cold storage in transplantation, providing notable benefits in graft preservation and repair. To better understand the current landscape of machine perfusion in Germany, we conducted a survey among all 39 liver and kidney transplant centers. A total of 22 centers (56%) responded, with 63% (14/22) reporting to have an active MP program. Liver transplantation programs utilize both hypothermic and normothermic perfusion, whereas perfusion strategies in kidney transplantation remain largely limited to hypothermic techniques. Most liver centers (57%; 8/14) apply MP selectively to marginal grafts. 43% (6/14) use it routinely for all accepted organs. Respondents reported an average 10% increase in organ utilization rates attributed to MP. While the clinical benefits of MP are widely acknowledged, key challenges persist, particularly regarding limited funding and insufficient clinical- and research infrastructure. The existing MP programs were predominantly financed through internal funding and only 33% (5/15) had a dedicated perfusion team. Current research in MP focus on viability assessment, objective graft evaluation criteria, organ repair, and strategies to expand the donor pool. Despite promising outcomes and increasing adoption, Germany needs a clear funding framework to fully integrate MP into routine clinical practice.

## Graphical Abstract

**Figure:**
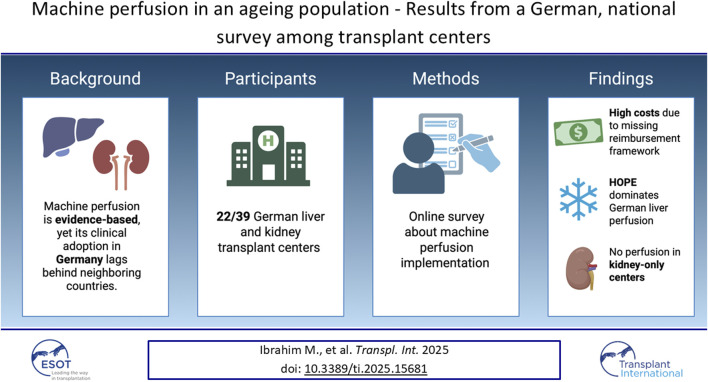

## Introduction

Organ transplantation of livers and kidneys is still considered the best and most cost-effective treatment option for end-stage organ failure [1]. The worldwide organ shortage continues to lead to high waiting list mortality rates [2]. Due to the growing discrepancy between supply and demand, the transplantation society worldwide is faced with the dilemma of accepting high risk grafts to enlarge the donor pool without jeopardizing the outcome of recipients [3]. In the context of an ageing population, both the donor and recipient demographics in transplantation are shifting. Increasing life expectancy and the rising prevalence of age-associated comorbidities result in a growing proportion of elderly patients on waiting lists, while organ donation is increasingly sourced from older donors, too. These donors are often with comorbid conditions such as hypertension, diabetes, or hepatic steatosis increasing the rate of extended criteria donor (ECD) grafts [4, 5]. These grafts are vulnerable to ischemia–reperfusion injury (IRI) resulting in inferior long-term outcomes if preserved by static cold storage alone [6, 7].

**FIGURE 1:**
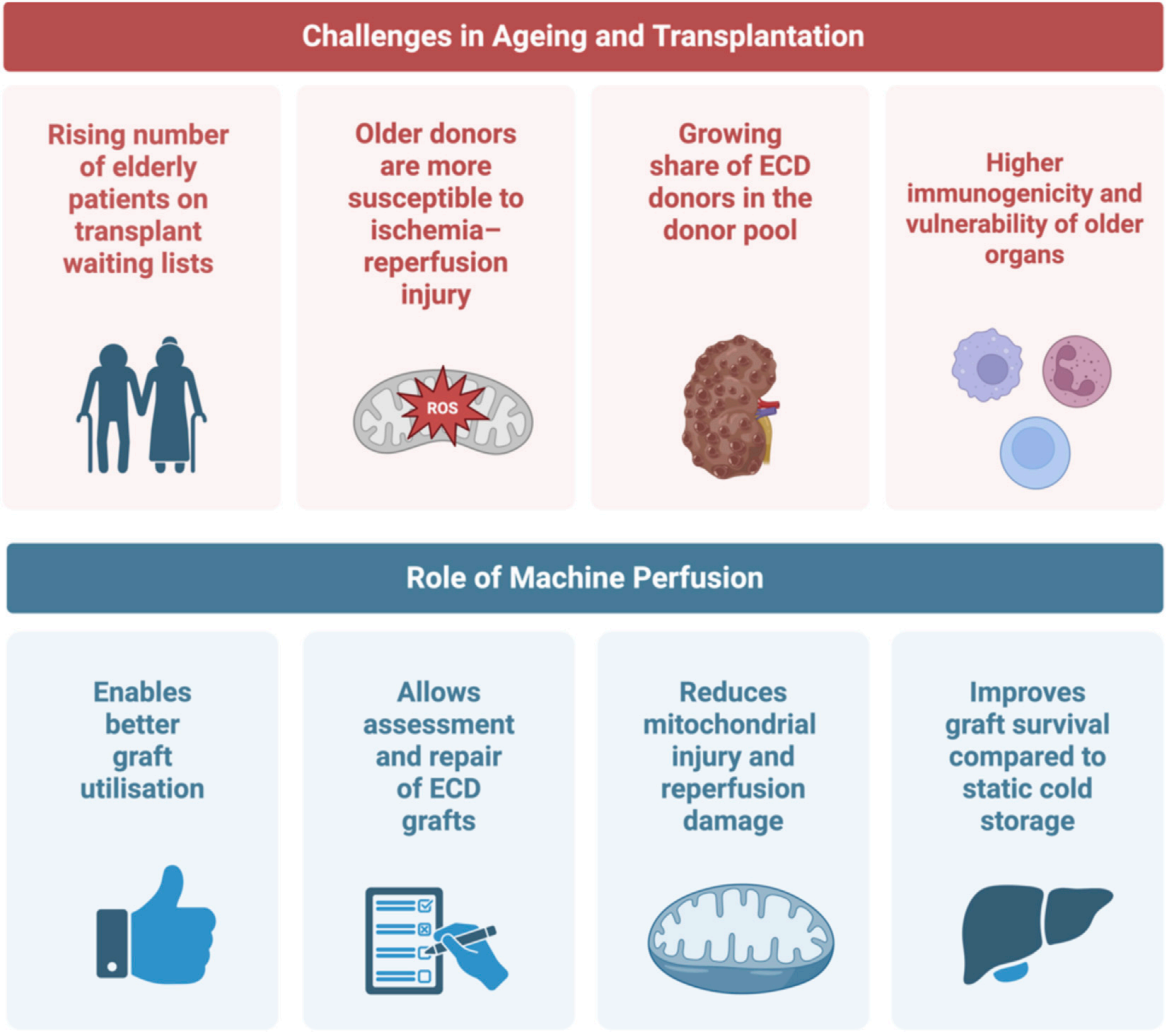
Transplantation challenges in an ageing population and the role of machine perfusion approaches as a potential solution (created in BioRender. Ibrahim, https://BioRender.com/mvkdmin).

Therefore, dynamic preservation strategies are continuously replacing static cold storage (SCS). Machine perfusion strategies offer the opportunity to repair, evaluate, and optimize organs prior to transplantation (Figure 1) [8]. As recently shown dynamic perfusion treatment even appears to attenuate the risks of transplanting elderly DBD livers and is linked to favorable long-term survival in ageing recipients [9]. Different modalities exist, with varying levels of evidence supporting their use in liver and kidney transplantation. In Europe, two machine perfusion techniques are currently most employed: hypothermic (oxygenated) perfusion (HMP, HOPE or D-HOPE) and normothermic machine perfusion (NMP). In HMP/HOPE, the organ is perfused at 8 °C–12 °C with an oxygenated or non-oxygenated acellular perfusion solution. In contrast in NMP the organ is kept at physiological temperature (37 °C) using an oxygenated, blood-based perfusate [10]. A combination of both approaches can be used, e.g., for controlled rewarming and viability assessment before transplantation [11].

### Machine Perfusion in Liver Transplantation

In liver transplantation, the evidence supporting hypothermic oxygenated machine perfusion (HOPE) as a superior preservation technique compared to static cold storage (SCS) is compelling [12]. Oxygenated hypothermic machine perfusion reduces ischemic-reperfusion injury through controlled hypothermic oxygen delivery. Rapid reperfusion of an organ usually leads to the release of toxic metabolites that accumulate in the mitochondria during the ischemia period. HOPE can counteract this effect by increasing ATP levels in the mitochondria and dialyzing toxic metabolites [13]. In contrast, NMP extends the concept of organ preservation under near-physiological conditions. NMP appears to enhance the utilization of grafts that might otherwise be discarded under SCS, likely due to the opportunity it offers for functional assessment of the organ [7, 14].

### Machine Perfusion in Kidney Transplantation

In the field of kidney transplantation, RCTs show superiority of hypothermic perfusion over SCS in terms of DGF in DCD and ECD-DBD grafts [15]. Preclinical studies in rodent models by Kron et al. have provided mechanistic insights into the protective effects of hypothermic perfusion. In kidney grafts, HOPE resulted in improved functional parameters compared to static cold storage. On the cellular level, perfused kidneys exhibited reduced nuclear damage, diminished activation of macrophages and endothelial cells, and attenuated rejection responses. These findings suggest that dynamic hypothermic preservation may confer immunomodulatory and cytoprotective effects that improve graft quality [13, 16, 17]. The clinical benefit of NMP in kidney transplantation remains unclear and has only been marginally investigated to date. An RCT from 2023 with 338 included patients did not show a significantly lower rate of DGF compared to SCS [18]. Overall, the available evidence supports the use of hypothermic machine perfusion as a superior preservation strategy for marginal kidney grafts.

### Machine Perfusion in Germany

Despite robust evidence supporting machine perfusion in liver and kidney, its clinical implementation in Germany is still evolving compared to several neighboring European countries. A well-known shortcoming is the lacking of DCD transplantation in Germany but the most recent German transplantation guidelines recommend the use of MP for marginal organs, reflecting growing recognition of its value, although it has not yet been established as routine clinical practice [19, 20]. A notable upcoming development is the nationwide implementation of hypothermic machine perfusion for all extended criteria donor (ECD) kidney grafts in Germany, using a portable perfusion device. This program, scheduled to begin in January 2026, is expected to significantly increase clinical utilization rates.

Here, we conducted a nationwide survey among German transplant centers to further understand the main obstacles preventing a routine implementation of machine perfusion in liver and kidney transplantation in Germany while considering the growing percentage of ECD grafts, e.g., grafts from octogenarians.

## Materials and Methods

An online survey on machine perfusion in liver and kidney transplantation in Germany was conducted between April 8, 2025 and April 25, 2025. For this purpose, all 39 German liver and kidney transplant centers were contacted via E-mail. A reminder E-mail was sent 1 week later to further increase the participation rate. The anonymous online survey contained 50 questions and was conducted using the survey platform surveymonkey.com. The questions covered center structure, clinical application of MP in liver and kidney, indications, as well as ongoing research projects on MP. In addition to multiple choice questions, free text responses were also included. The survey was approved on March 20, 2025 (project no. 026/2025BO2) by the Ethics Committee of the Medical Faculty of the University of Tübingen. The statistical analysis is descriptive. Continuous variables were summarized using medians and averages, while categorical data were described using counts and percentages. Percentages were rounded to the nearest whole number.

## Results

### Transplant Center Characteristics

22/39 (56%) of the centers contacted took part in the online survey, of which 15/19 (79%) were liver transplantation centers and 22/37 (60%) were kidney transplantation centers. 15/22 transplant centers surveyed transplanted both livers and kidneys. Of the 22 kidney centers, four centers transplanted kidneys and pancreas and three clinics only kidneys. On average, 44 liver transplants are performed per center and year with the highest reported number of 75 transplants/year. The average for kidney transplants was 57 among the 22 centers surveyed, ranging from 29 to 130 operations per year. Pediatric transplants were performed by 12/22 centers (55%). Transplants with living kidney donations were performed by 21/22 (95%) centers included, 10/15 (67%) liver centers reported transplants with living liver donations Table 1.

**TABLE 1:** Center structure, funding, and research.

How many liver transplant centers?	15	
How many kidney transplant centers?	22	7 of them perform only kidney +/- pancreas
Average number of liver transplants per year	44	
Average number of kidney transplants per year	57	
Are living donor transplantations performed?		
Living donor liver	10/15 (67%)	
Living donor kidney	21/22 (95%)	
Are pediatric transplantations performed?		
Yes	12/22 (55%)	
No	10/22 (45%)	
Do you have a machine perfusion (MP) program?		
Yes	14/22 (63%)	
No	7/22 (32%)	All of them kidney-only centers
No, but planned	1/22 (5%)	
What type of perfusion do you use?		
NMP	3/15 (20%)	
HMP/HOPE	7/15 (47%)	
Both, depending on indication	5/15 (33%)	
Do you have a dedicated perfusion team?		
Yes (≥3 members)	5/15 (33%)	100% physician-led
No	10/15 (67%)	
How is the MP program funded? (Multiple answers possible)		
Hospital funds	14/14 (100%)	
Research grants	6/14 (43%)	
Other	1/14 (7%)	Study funding
What is the greatest barrier to establishing an MP program?		
Cost	10/20 (50%)	
Personnel demand	7/20 (35%)	
Lack of evidence	2/20 (10%)	
Lack of expertise	1/20 (5%)	
Is there an MP research group?		
Yes	11/19 (58%)	
No	8/19 (42%)	
What are your research objectives? (Multiple answers possible)		
Expansion of donor pool	14/16 (87%)	
*Ex-vivo* organ repair	15/16 (93%)	
Organ enhancement	15/16 (93%)	
OR team relief	6/16 (38%)	

### Machine Perfusion Characteristics

14/22 (63%) of the clinics surveyed have an established MP program. All centers that perform liver transplants stated that they already perform machine perfusion or plan to do so in the future. Of the seven centers that only transplant kidney (+/- pancreas), none of them had an established perfusion program. 11/13 (85%) of the programs were established between 2020 and 2024. Only 5/15 (33%) of centers reported having a perfusion team with ≥3 employees. The majority of clinics surveyed stated that they perfuse hypothermic (7/15; 47%). 3/15 (20%) of respondents stated a preference for normothermic perfusion, while 5/15 (33%) of the centers used both approaches depending on the indication. All respondents financed their perfusion via internal hospital funds. 7/14 (50%) used research and study funds. The majority of respondents (10/20; 50%) saw costs as the biggest barrier to use, followed by personnel demand (7/20; 35%). 2/20 centers (10%) cited the lack of evidence as the main barrier. 9/14 (64%) stated an increase of organ utilization due to machine perfusion. The average, self-reported increase in organ utilization since the introduction of an MP program was estimated 5%–30%. 12/14 (86%) of respondents stated that they perform transplants at off-peak times despite machine perfusion. The promotion of young surgeons through MP was denied by 10/14 (71%) of respondents. 1/14 (7%) MP programs stated that they had rejected an organ due to technical problems occurring during MP. All respondents stated that they see a clear advantage for German transplant programs in MP (Table 2). The main focus of the respondents in MP research was in the fields of organ improvement and expansion of the donor pool. 11/19 (58%) transplant centers had a research group further investigating MP.

**TABLE 2:** Indication, rejection rate and organ utilization with MP.

Does MP provide an advantage in transplantation?	
Yes, always	11/14 (79%)
Yes, but only for ECD organs	3/14 (21%)
No	0/13 (0%)
Has MP led to increased organ utilization?	
Yes	9/14 (64%)
No	5/14 (36%)
If yes, by how much?	
0%–10%	8/13 (62%)
11%–20%	3/13 (23%)
>20%	2/13 (15%)
Are transplantations performed at other times due to MP?	
Yes	2/14 (14%)
No	12/14 (86%)
Does MP support the training of young surgeons?	
Yes	4/14 (29%)
No	10/14 (71%)
Has an organ been declined after MP for technical reasons?	
Yes	1/14 (7%)
No	13/14 (93%)

### Use of Machine Perfusion in Liver Transplantation

Among the 14 liver centers surveyed, 792 liver transplants were performed using machine perfusion in the last 5 years in Germany. Almost all of the liver centers participating stated that they had an MP program. Only one center was currently planning an MP program. 9/14 (64%) of the centers used HOPE and 11/14 (79%) perfused between 2 and 4 h. While only a few centers (3/14; 21%) performed viability testing during HOPE, lactate clearance (9/10; 90%) and bile production (6/10; 60%) were considered valid viability criteria for NMP. While there was agreement on the advantages of MP, there was disagreement on the indication for MP. When asked about the indication for liver MP, 6/14 (43%) of liver centers stated that they perfuse all accepted organs, while 8/14 (57%) primarily perfused marginal organs (Table 3).

**TABLE 3:** Clinical use of MP in liver and kidney transplantation.

Questions	Liver transplant	Kidney transplant
How high is your rejection rate after organ allocation?		
0%–15%	5/12 (42%)	9/10 (90%)
16%–30%	4/12 (33%)	0/10 (0%)
>30%	3/12 (25%)	1/10 (10%)
Average	25%	11%
What is the most frequent reason for non-transplantation?		
Liver Macroscopy	7/14 (50%)	-
Steatosis in biopsy	7/14 (50%)	-
Kidney Macroscopy	-	5/9 (56%)
Suboptimal graft perfusion	-	3/9 (33%)
Other	-	1/9 (11%)
Which type of MP is used?		
HMP/HOPE	9/14 (64%)	6/7 (86%)
NMP	3/14 (21%)	0/7 (0%)
HMP/HOPE and NMP equally	2/14 (14%)	1/7 (14%)
HMP/HOPE and NMP sequentially	0/14 (0%)	0/7 (0%)
Dual perfusion	5/14 (36%)	-
Portal only	7/14 (50%)	-
Dual und portal equally	2/14 (14%)	-
How many hours of perfusion?		
1–2 h	-	1/5 (20%)
2–4 h	11/14 (79%)	2/5 (40%)
>4 h	3/14 (21%)	2/5 (40%)
Viability assessment during HOPE?		
No	11/14 (79%)	4/5 (80%)
Yes	3/14 (21%)	1/5 (20%)
Viability criteria for NMP? (Multiple answers possible)		
Lactate clearance	9/10 (90%)	-
pH >7,30	5/10 (50%)	-
Bile production	6/10 (60%)	-
Bile pH >7,45	3/10 (30%)	-
Glucose metabolism	4/10 (40%)	-
Urine production	-	2/4 (50%)
Kidney resistance	-	3/4 (75%)
Base excess in blood gas analysis	-	2/4 (50%)
Indications for MP? (Multiple answers possible)		
ECD organs	8/14 (57%)	6/7 (86%)
Expected long ischemia time	7/14 (50%)	4/7 (57%)
Donor age >65	5/14 (36%)	1/7 (14%)
All transplantable organs perfused	6/14 (43%)	0/6 (0%)
Advantages of MP? (Multiple answers possible)		
Organ protection	13/14 (93%)	6/8 (75%)
Reduced reperfusion injury	14/14 (100%)	7/8 (88%)
Extended monitoring	6/14 (43%)	3/8 (38%)
Logistics	7/14 (50%)	-
How many organs have been perfused in the last 5 years?		
0–50	8/14 (57%)	9/9 (100%)
51–100	5/14 (36%)	-
>100	1/14 (7%)	-
Total	792	47

### Use of Machine Perfusion in Kidney Transplantation

41% (9/22) of the kidney centers surveyed stated that they had performed kidney perfusion in the last 5 years. All 9 centers also transplanted livers at their center. All 7 centers surveyed that solely transplant kidneys (kidney only) stated that they do not have an MP program. Thus, the total number of perfused kidneys among the respondents in the last 5 years is 47. 6/7 (86%) prefer hypothermic perfusion for kidney transplantation. For 6/7 (86%), the main indication for perfusion was ECD organs. 7/8 (88%) saw an advantage of MP in terms of reduced reperfusion damage (Table 3).

## Discussion

Our survey revealed a heterogeneous yet increasingly structured implementation of machine perfusion across German transplant centers. While 63% of centers reported having an established MP program, its use remains predominantly limited to liver transplant centers, with kidney-only centers rarely engaging in MP. Hypothermic (oxygenated) perfusion is the most applied modality for both liver and kidney grafts. In liver transplantation, most centers use HOPE for marginal organs, while normothermic perfusion is used selectively in cases with the need for viability testing, with lactate clearance and bile production cited as the most relevant functional markers. In kidney transplantation, the overall use of MP remains low, and is primarily reserved for extended criteria donor (ECD) grafts. Barriers to implementation include cost, personnel demands, and lack of dedicated perfusion teams. Despite these challenges, nearly all respondents recognized the potential of MP to improve organ utilization rates and the expansion of the donor pool. Self-reported organ utilization rate averaged 5%–30%, with 9/14 (64%) centers stating an increase of its department utilization rate due to machine perfusion. Despite existing infrastructure and a generally high level of motivation for implementation, the actual organ perfusion activity in Germany remains limited. According to self-reported estimates, only 792 liver grafts and 47 kidney grafts were perfused across all participating centers over the past 5 years. With a total number of 3910 DBD liver transplants (2020–2024, DSO data) according to the German Organ Procurement Organization (DSO), one fifth of the livers are currently perfused. The situation is different in the field of kidney transplantation. Organ perfusion only plays a role in centers that also have a liver transplant program. The respondents from the “kidney-only centers” denied the use of machine perfusion for transplantation. Out of 7364 DBD kidney transplants in Germany from 2020 to 2024 (DSO data) only 47 kidneys were perfused in the last 5 years (0.6%) [21].

### Biggest Barriers - Costs and Personnel Demands

Healthcare costs represent a critical factor influencing clinical decision-making and the adoption of new technologies in medical practice. A major barrier to the widespread implementation of machine perfusion is the associated cost and personnel demand. The devices, including disposables and setup, relate to substantial expenses, often amounting to several tens of thousands of euros per case. Furthermore, perfusion requires dedicated personnel to prepare, monitor, and manage the system throughout the procedure. Hypothermic perfusion of the liver is reported to create approximately 5000€ more cost than SCS/case [19, 22]. Normothermic liver perfusion is estimated at 15,000 USD/case according to Raigani et al. [23]. These logistical and financial challenges greatly diminish the feasibility of routinely implementing such technologies, especially in centers with limited resources, staffing shortages, and no reimbursement framework in place by the health insurance system in Germany. Hypothermic machine perfusion of kidney grafts has been shown to be cost-effective already. An economic evaluation of a multicenter RCT comparing machine perfusion with static cold storage mainly in the Netherlands demonstrated that MP is both cost-effective and clinically superior across deceased donor kidney transplants, particularly in extended criteria donors. MP was associated with lower first-year costs, higher graft function rates, and improved long-term survival [24].

Similar cost-saving effects have been observed in DCD liver transplantation. Over the first post-transplant year, perfused grafts were associated with a 12.2% reduction in overall costs, primarily due to shorter ICU stays and reduced complication rates. When accounting for the additional expenses of the perfusionist and machine, cost savings were still evident and estimated to offset the investment at a volume of approximately 25–30 liver transplants per year [25]. The German RCT by Czigany et. al. from 2021 showed a reduced intensive care and hospital stay compared to SCS, as well as lower complication rates following livers being perfused [19]. The existing evidence clearly indicates that the costs per perfusion are cross covered by a better outcome and faster patient recovery. Furthermore, the higher utilization rate of organs, may lead to an expansion of the donor pool and reduction of waiting list time as well as reduction of waiting list mortality. Existing evidence supports the hypothesis that earlier transplantations can reduce costs [23, 26].

**FIGURE 2:**
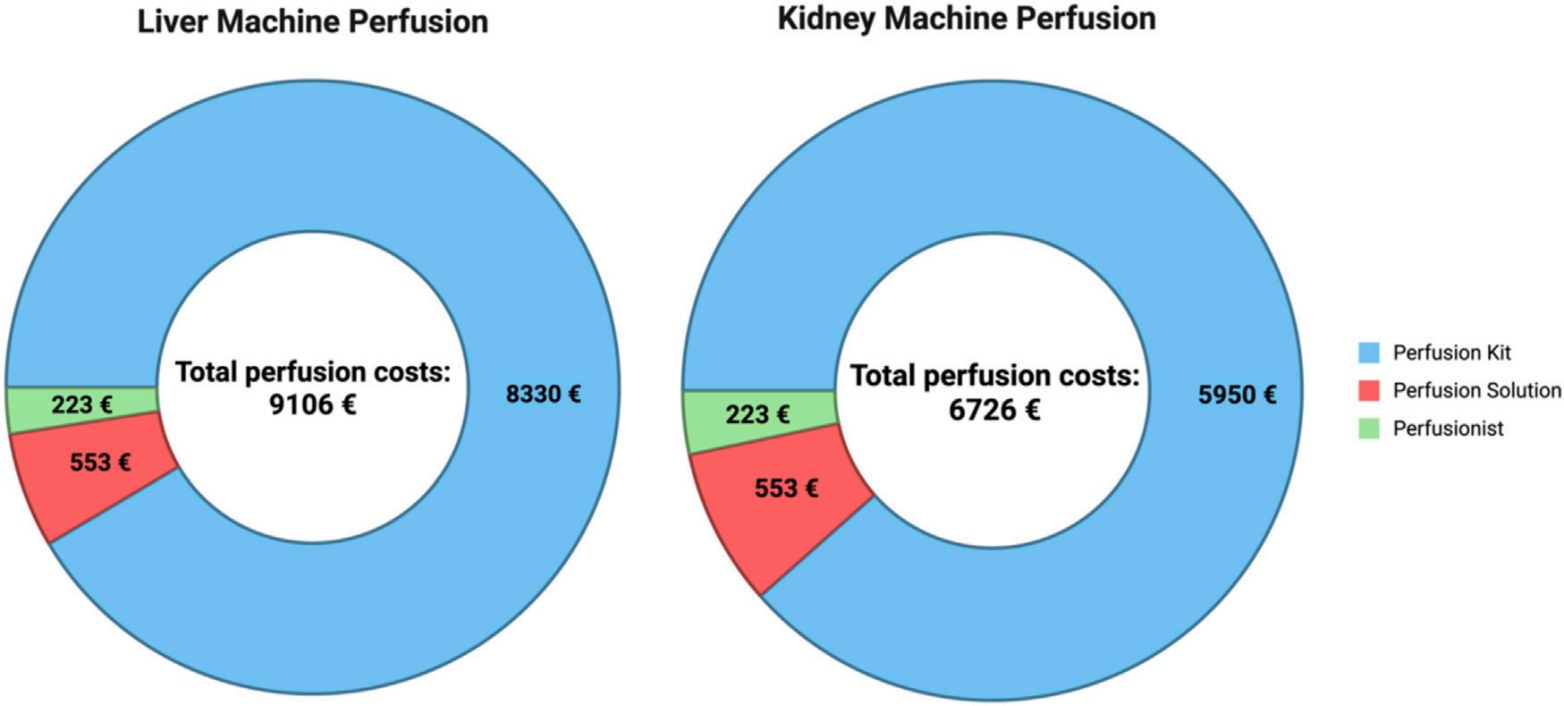
Average costs (EUR) per case for hypothermic liver or kidney machine perfusion at the Surgical Department, University Hospital Tübingen. Cost data are collected by our internal controlling department. Perfusion costs are currently not reimbursed by the German health insurance system and are solely covered by internal hospital funding. (created in BioRender. Ibrahim, https://BioRender.com/lv1soun).

A detailed cost analysis from Germany to further investigate this highly relevant topic has to follow. If there is sufficient evidence, costs must be covered by the local health insurance systems to achieve broader implementation of machine perfusion with its advantages in Germany. The broader use in the Netherlands, for example, can be explained by the financial coverage of perfusion approaches in DCD organs by state institutions [26]. In this survey, it is particularly clear that smaller programs (kidney transplantation only centers, low organ transplantation rates) do not have perfusion programs. This is certainly due to the potentially connected high costs and personnel requirements. To illustrate the financial burden, an overview of perfusion costs from our department, collected by the controlling unit of the University Hospital Tübingen, is presented in Figure 2. Machine perfusion generates additional costs of 9,106 € per liver and 6726 € per kidney, which are solely covered by internal hospital funding.

The lack of personnel was also frequently cited as a major barrier for broader implementation in the survey. Unlike simple cold storage, machine perfusion requires trained staff. Most centers lack a dedicated perfusion team, as reflected in the survey results. Consequently, transplant surgeons or medical students are often assigned to operate the perfusion devices. It is of utmost importance to create and implement a structured organ perfusionist curriculum in clinical routine like in the field of cardiovascular surgery. In 2022, the first educational program for organ perfusionists was launched in the Netherlands, offering structured training that includes lectures on organ physiology and hands-on practice with perfusing livers, kidneys, hearts, and lungs [27].

The image of the well-rested transplant surgeon is often associated with dynamic organ preservation. In addition to improved organ preservation, machine perfusion is intended to make transplantation an “semi-elective”, schedulable, less time-critical operation. In this way, operations might to be carried out during daytime. Furthermore, the broader implementation of machine perfusion strategies may facilitate the training of the next-generation of transplant surgeons. So far, this is not the reality in Germany. According to the survey, organ transplants are neither transferred to the elective program, nor are younger colleagues trained more often because of MP. On the contrary, due to the higher personnel demand and the scarcity of perfusion teams, there is presumably a higher pressure on the team involved in transplantation with all the logistics.

### Evidence Follows Clinical Implementation - Indication and Viability in Machine Perfusion

For the successful implementation of novel technologies in clinical practice, clear guidelines, robust evidence from randomized controlled trials, and support from national reimbursement frameworks and industry partners are essential [28]. Our survey highlights that significant uncertainty remains regarding viability assessment and the clinical indications for perfusion in both liver and kidney transplantation.

To date, six randomized controlled trials (RCTs) on hypothermic oxygenated perfusion (HOPE) and four RCTs on normothermic machine perfusion (NMP) in liver transplantation have been published. A recent meta-analysis by Patrono et al., which also included five cohort studies, demonstrated that HOPE is associated with a reduced incidence of ischemic cholangiopathy (IC) and improved graft survival in both DBD and controlled DCD liver transplantation [12]. In contrast, NMP did not show significant improvements in either IC or graft survival. The first RCT on NMP, conducted by Nasralla et al., reported a significant reduction in peak AST levels within the first seven postoperative days and a lower organ discard rate, though IC and graft survival rates were comparable to those with static cold storage [14]. Two subsequent RCTs reported similar findings [29, 30]. Notably, the only RCT to demonstrate a significant reduction in early allograft dysfunction (EAD) following NMP was the study by Markmann et al. [31]. Supporting this, a subgroup analysis by Chapman et al. showed lower EAD rates in DCD liver grafts preserved with NMP [30]. To date, no randomized controlled trials have directly compared normothermic machine perfusion (NMP) and hypothermic oxygenated perfusion (HOPE), which poses a significant limitation for the development of national guidelines and the establishment of standardized clinical protocols [27].

Sequential perfusion, combining hypothermic oxygenated perfusion (HOPE) followed by normothermic machine perfusion (NMP), has been explored by groups in Groningen and Cleveland to leverage both the protective effects of HOPE and the viability assessment capabilities of NMP. This approach has primarily been applied to organs with unfavorable donor profiles. While the technique is considered safe and technically feasible, its clinical benefit remains uncertain [32, 33].

In kidney transplantation, a recent meta-analysis including 22 RCTs by Tingle et al. demonstrated that hypothermic machine perfusion (HMP) significantly reduces the incidence of delayed graft function (DGF) and improves graft survival in both DCD and extended criteria DBD kidneys [15]. However, the use of DGF as a primary endpoint is subject to ongoing debates, as it does not necessarily correlate with poorer long-term outcomes [34]. Moreover, the impact of non-oxygenated HMP on other clinically relevant outcomes—including primary non-function (PNF), acute rejection, patient survival, hospital length of stay, long-term graft function, and duration of DGF—remains uncertain [15].

Guideline development should account for the fact that regulatory, ethical, and clinical practices surrounding organ procurement and transplantation vary significantly between countries. In Germany, where DCD transplantation is currently prohibited, the use of kidney perfusion remains limited. This is not surprising, given that most of the available evidence supporting machine perfusion pertains primarily to DCD grafts. Notably, the German Society of Nephrology has expressed strong reservations about implementing a nationwide perfusion program for ECD kidneys, citing the lack of prospective data supporting its benefit in DBD donors [35].

In addition to improved preservation, one of the key objectives of machine perfusion is to expand the donor pool by enabling the transplantation of organs that would otherwise be discarded. A central argument among proponents of machine perfusion is its potential for viability assessment. In our survey, transplant centers reported using various markers to assess viability [36]. Notably, most centers using hypothermic oxygenated perfusion (HOPE) did not perform viability assessment, whereas normothermic machine perfusion (NMP) was frequently used for this purpose. The most used biomarkers in NMP were lactate clearance (90%), pH-hemostasis parameters (50%), and bile production (60%) in liver perfusion, as well as vascular resistance and flow parameters (75%) in kidney perfusion.

In HOPE, the Zurich group identified flavin mononucleotide (FMN) as a promising biomarker for organ viability. FMN is released upon mitochondrial complex I injury and has shown a strong correlation with coagulation factors and peak transaminase levels after liver transplantation [37, 38]. FMN was recently validated in a normothermic machine perfusion (NMP) cohort, where elevated perfusate FMN levels (>1.75 μg/mL at 4 h) were independently associated with reduced graft survival and death-censored graft survival [39]. These findings support FMN as a robust viability marker in both HOPE and NMP settings.

During normothermic machine perfusion (NMP), the organ remains metabolically active, as it is perfused at physiological temperatures. The liver plays a key role in lactate clearance through hepatocellular oxidation and gluconeogenesis [36]. While several studies have shown an association between lactate metabolism and postoperative liver function, lactate alone is considered an unreliable marker of viability. Most livers are capable of clearing lactate during NMP, and even limited hepatic function may be sufficient to normalize lactate levels in the relatively small volume of perfusate used. Therefore, it is recommended to apply composite viability criteria that include additional markers such as perfusate pH, glucose levels, and transaminase concentrations, as reflected in most current NMP studies [40–43].

To date, there is no clinical consensus on which parameters or thresholds are most reliable for assessing viability. Identifying clinically relevant viability markers remains challenging, as organs deemed non-viable cannot be ethically transplanted for outcome validation. This ethical limitation, combined with the heterogeneity of perfusion protocols, including differences in temperature, duration, perfusate composition, oxygenation strategies, and additives such as bile salts, insulin, or bicarbonate, further complicates standardization and interpretation of viability data [36].

### Future of Machine Perfusion in Europe - Guidelines and Perfusion Hubs

The use of machine perfusion in organ transplantation is expanding globally, with clinical implementation progressing rapidly across Europe, including Germany. Our survey revealed that 85% of MP programs were established between 2020 and 2024, highlighting MP as one of the most significant developments in the field of transplantation in recent years [28]. Consensus documents, such as the newly published ELITA guidelines, may facilitate harmonized implementation across European centers [44]. However, standardization is still hindered by a lack of multicenter randomized controlled trials, particularly those directly comparing HOPE and NMP in liver transplantation. Future efforts should focus on advancing preclinical and clinical research on viability assessment, which holds promise for expanding the donor pool, especially in liver transplantation. The development of centralized perfusion hubs for research, organ optimization, and viability testing could serve as a pan-European initiative to enhance perfusion-based organ preservation. Additionally, establishing national and international registries and implementing standardized perfusion protocols would improve comparability of outcomes and data quality [28]. Integrating composite viability scores with AI-based predictive models may further support clinical decision-making. Our survey provides insights into current challenges and priorities for MP implementation from a German perspective. Further surveys conducted across Europe and globally are essential to identify region-specific needs and facilitate targeted, context-sensitive strategies for successful integration of MP into clinical practice.

## Conclusion

Both the donor pool and the recipient cohort are increasingly characterized by advanced age and multimorbidity with all its connected disadvantages [6]. Machine perfusion has demonstrated protective effects in elderly grafts by stabilizing mitochondrial function and reducing reperfusion injury, ultimately improving outcomes [13]. For a nationwide routine implementation, a reimbursement framework within the German health insurance system is urgently needed. This is of particular importance, as many other European countries have already implemented such structures, and robust evidence demonstrates the clinical benefit of machine perfusion.

## Data Availability

The raw data supporting the conclusions of this article will be made available by the authors, without undue reservation.
